# Treatment of the Teeth during Pregnancy

**Published:** 1890-02

**Authors:** W. H. Dwinelle

**Affiliations:** New York City


### International Dental Journal.

VOL. XI. FEBRUARY, 1890. No. 2.

### Original Communications.[1]

### TREATMENT OF THE TEETH DURING PREGNANCY.[2]

BY W. H. DWINELLE, M.D., D.D.S., NEW YORK CITY.

THE mere mention of pregnancy suggests to the mind of all a deranged and, in a certain sense, an abnormal condition of the system involving acrid and acidulated secretions of the alimentary canal, especially of the mouth. Before proposing a system of treatment for teeth during pregnancy, we will consider their condition under this contingency, the *cause* of that condition, and then the remedy. The causes may be (1) local and (2) constitutional,—the nervous derangement of the system and the profound constitutional and mental disturbance.

In a pregnant woman we recognize a human being whose function and office is to reproduce within herself another living being. She is to supply from the alembic of her own ever-accumulating and ever-wasting storehouse the material to be modified and assimilated into a duplication of herself. All the elements must be appealed to to contribute to build up and perfect the organization in her charge; especially must she furnish an abundance of the

[1] The editor and publishers are not responsible for the views of authors of papers published in this department, nor for any claim to novelty, or otherwise, that may be made by them. No papers will be received for this department that have appeared in any other journal published in this country.

[2] Read at the Nineteenth Annual Session of the New Jersey State Dental Society, Asbury Park, July 19, 1889.

elements of the phosphate and carbonate of lime for the bones and teeth of the embryo man. These elements must be supplied and derived from the food taken into the system by the mother. If there is not a sufficient supply furnished from this source, it must, to a certain extent, be taken indirectly from her own organization and appropriated to that purpose. So that her system, being robbed at large of the elements needed for the child, literally feeds on itself, like Dr. Tanner, fasting for forty days, consuming seventy pounds of himself, or like bears hibernating,—the winter cannibals!

We do know that we are but chemical laboratories, containing all of the elements within ourselves. Knowing this, it is easy to conceive how these elements may be constantly changing their relation to each other, how they may, by chemical resolution, be brought into solvent condition and be appropriated or diverted from one part of the system to another. To pursue the subject of waste, absorption, and appropriation further, it may be possible that in the process the tax on the tooth-material is not alone called upon, but that the bones generally have to yield a portion of their lime salts, even to the loss of their integrity for the time, to satisfy the demands of nature. We have no doubt that rickets and bone-malformation generally might be traced to deficiency of lime at the critical period when it was most needed.

The craving or longing of pregnant women for lime, for chalk, plaster, and clay are indicative of this demand of nature. Hens will not lay eggs with perfect shells unless they have access to lime in some form, and every aviary has for its motto, "No lime, no eggs; no eggs, no birds." The wild animals of the woods make pilgrimages to the "deer lick" to abate their longing for salt; all of these, and scores of others, are but dumb appeals from the unsatisfied chemistry of nature.

Assuming that we have a full appreciation of all the unhappy environments and associations connected with pregnancy, the exalted nervous tendencies, the mental and physical disturbances, the acrid and irritating secretions that come of them, the appeals to our higher sympathies and loving and tender care, giving the fullest meaning to the sentiment that the gentlest natures, when shattered by incessant pain, enfeebled by disease, often misrepresent themselves and permit the spasmodic throes of a pain-goaded organization to be the exponent of what *they are not.* Admit all these things, and assuming every sweet sentiment in tribute to "God's best gift to man," the practical question is still before us,—*the treatment of the teeth during pregnancy.*

The first thing and the last that meets us at the very threshold of our investigation is acids and acrid secretions, dissolving and destroying the teeth, accompanied with pain and exalted sensitiveness, to a degree beyond expression. I have repeatedly observed instances where patients' teeth have decayed and wasted more during a single period of gestation than during all their lives before.

*What is the remedy and what the treatment?*

The first thing to be done is to consider the importance of the general nutrition of the mother with well-selected diet, and especially whole wheat food, to make sure of the phosphate portion of them which is contained in the four outer capsules of the kernel, and which in the manufacture of superfine flour are wholly rejected. Oatmeal, too, is an excellent phosphate cereal. The next thing is to charge the system, through the stomach, with bone phosphate of lime in solution or in the form of a powder, spreading the same upon the food to be eaten, or distributing it over the same with a receptacle in the form of a pepper-box, literally using it as a diet, until the system is *saturated* with it; at the same time, it will be well to use lime-water, natural Vichy water, ever dieting towards the *alkaline.*

Use tooth-powder in which bicarbonate of soda is a generous ingredient, and have all washes of the mouth contain the same. For general tonics I use sometimes other forms of the phosphates, such as the hypophosphates and lactophosphates, with excellent results. Under some conditions I use nux combined with myrrh and tincture of iron; this last, when used with Vichy water, is entirely harmless to the teeth.

In using the phosphates, select that which is derived from the bones of animals, never that from the phosphate rock; the bone-phosphates are potent and are readily taken into and assimilated with the system. The phosphates from the rock are inert, extraneous, non-assimilable, and useless to our animal economy, although chemically they are the same.

For acid and sensitive teeth, subdue with soda applied in saturated solution to the teeth, then with such obtunders as may be most effective; excavate and fill with pink gutta-percha to bridge over to the period when the patient is able to endure more permanent operations.

Extracting the teeth during pregnancy should be avoided, if possible; try such palliative treatment as may bring the teeth into subjection; but if this cannot be done, and the pain from them becomes hazardous, do not hesitate to remove them.

We know that we can change to a great degree the density of the soft structure of the teeth of young, overgrown girls—who, by overstudy, under-feeding, and under-sleeping, have rendered them so—in a few months by proper mental and physical rest and phosphate treatment; for we have ourselves done this repeatedly to the welfare and safety of the teeth which otherwise would have been lost, giving us assurance that in the ever-changing chemistry of the varying solvents of our organization they may be so directed that the problem of recalcification will be capable of demonstration and subject in a large measure to our control. I forgot to mention, in passing, that none of the cereals is richer in phosphates than Indian corn. Barlow, one of our New England poets, referring to the sturdiness of the men of his time, said of them,—

"Whose bones are made of Indian corn!"

I might refer to phosphate paste and my method of using it, but time forbids. I might refer to a case reported several years ago, wherein rapid erosion of the teeth was immediately arrested by its use, together with general treatment, to general absorption, and wasting of the teeth; but this would come under another head, except so far as it might illustrate the theory of the diversion of the elements of our system.

I copy from my note-book two cases which, with many others, confirm in my mind all I have said in favor of the bone-phosphate of lime.

"Mrs. C. had passed through two painful periods of gestation, with excessive acid secretions and vomiting most of the time, giving birth to a girl ten years ago, and two years later to a boy. The girl's first teeth were remarkably poor. All that were erupted of the second set can be cut away as though they were chalk. The teeth of the boy are all even worse than those of the sister."

When the mother became pregnant a third time, with all the symptoms in an unusually aggravated form, I at once put her on the phosphate treatment, and prescribed diet together with general tonics. She at once began to mend. The acid secretions and eructations abated entirely, her vomiting ceased at the end of the second month, and did not return. She passed through a remarkably healthy gestation, free from all annoyance or discomfort, when she gave birth to a boy. You may anticipate that I watched the eruption of his first teeth with interest. They proved to be of the finest quality. The six-year teeth are already fully erupted, perfect in all respects, and of unusual density.

Another case, which I will not give in detail, wherein a mother had four children. With the first there was no phosphate treatment; child's teeth very poor. In the second phosphate treatment; the best of teeth, second set most all in. Third child, mother away in Europe, no treatment; very poor teeth, both sets. Fourth child, phosphate treatment; the best of teeth so far. I could refer to many other cases confirming the benefits of phosphates.

As regards the primitive phosphate rock, it is unassimilable and inert to the animal economy. It had to go through a process nearly commensurate with the age of the world up to a comparatively recent period,—a process involving its passing from its primitive state through the stomachs of animals, ever evolving higher and higher through the ages till it reached the kindred mammalia, the phosphates of whose bones alone are potent and assimilable to the human system, and yet each are chemically the same; showing that there is a subtle chemistry behind, which the chemistry of our boyhood takes no cognizance of.

The vegetable kingdom cordially responds to the tonic influence of animal phosphates, as all agriculturists and fruit-growers know.

The theme of the ever-changing chemistry of our systems could be enlarged upon to the extent of a very interesting essay. I hope some will avail themselves of the opportunity of distinguishment in this direction.

I do not claim any special originality in what I have laid before you. Others have given their experience and successes, for which I feel under great obligations to them. I attempt only to give you my own and my faith in the promise it gives.

In the future I believe that the preparations of the animal phosphates are to take an important place in our practice. With children as well as those of maturer years its influence for good is marked and decisive, as we have already demonstrated. Pregnant women thrive on it, nature responds to it with avidity, and the most happy results follow its use. Our profession more than any other is interested in its quality and its potency to overcome many of the evils that beset us, which I believe we shall yet accomplish to the relief of humanity and the glory of our calling.

